# Integrating Care Context With Skeleton and Depth Information for Older Adult Activity Recognition in a Care Facility Using Care-Assessment-Aware Spatiotemporal Transformer: Method and Validation Study

**DOI:** 10.2196/80102

**Published:** 2026-04-02

**Authors:** Nazmun Nahid, Iqbal Hassan, Md Atiqur Rahman Ahad, Sozo Inoue

**Affiliations:** 1 Kyushu Institute of Technology Kitakyushu Japan; 2 University of East London London United Kingdom

**Keywords:** older adult activity recognition, activity recognition, care data, older adult dataset, transformer.

## Abstract

**Background:**

Older adult activity recognition is a critical task in long-term care monitoring; yet, it remains challenging due to postural deformities and health-related variability. These factors cause different activities to appear visually similar, or the same activity to appear dissimilar, undermining the effectiveness of traditional human activity recognition models developed for the general population.

**Objective:**

This study aims to develop an improved older adult activity recognition method that integrates care assessment information with motion data to capture and understand movement variability arising from different health conditions.

**Methods:**

To achieve our objective, we propose a care-assessment-aware spatiotemporal transformer (CSTT) model that integrates body key points, heatmaps, and care level data for personalized and context-aware activity recognition. The model dynamically adjusts its attention mechanism based on care level context to improve recognition accuracy. CSTT was trained and validated on real-world older adult motion data. A total of 51 older adult participants (30 men and 21 women; age range of 64-95 years) were included in the study. Among them, 7 (13.7%) required high care assistance, 26 (51.0%) required medium care assistance, and 18 (35.3%) required low care assistance.

**Results:**

Despite data imbalance and considerable intraclass variation due to differing care needs, the proposed CSTT model achieved an *F*_1_-score and accuracy of 0.96 and area under the curve is 0.98. Analysis revealed that movement patterns differ significantly across care levels and that similar motions occur in distinct activities, highlighting the importance of care-aware modeling.

**Conclusions:**

Incorporating care level information into activity recognition models significantly enhances performance in older adult care settings. The proposed CSTT framework demonstrates the value of personalized, context-sensitive approaches for accurate and ethical monitoring in long-term care environments.

## Introduction

### Background

Advancements in medical science and technology have led to a global demographic shift, with the older adult population projected to reach 1.2 billion by 2025 and 2 billion by 2050 [1]. By 2050, nearly 20% of the world’s population will be older adults [2]. As life expectancy increases, age-related physical and cognitive decline necessitates long-term care (LTC) [3], placing significant strain on health care systems and LTC facilities [4-8].

Caregivers in LTC facilities perform essential tasks, including hygiene assistance, feeding, dressing, and mobility support [7-10], while also providing emotional and psychological care [11]. However, their close interaction with residents exposes them to emotionally taxing experiences, such as witnessing chronic pain, cognitive decline, and end-of-life care [12], leading to compassion fatigue and emotional exhaustion. Caregivers also navigate ethical dilemmas, balancing residents’ autonomy with safety, managing conflicts with families, and making complex decisions [13-15]. These challenges heighten stress and contribute to burnout [16-18], exacerbating the global caregiver shortage, particularly in Central Asia and Eastern Europe. High annual turnover rates (19%-55%) [19-21] worsen staff shortages, increase workloads, and reduce care quality [22]. To address shortages, LTC facilities increasingly rely on health care assistants and auxiliary nurses [23]. While vital, their limited training can hinder the care of residents with cognitive impairments or chronic conditions [24], affecting both caregiver well-being and overall health care sustainability. Ensuring caregiver well-being while maintaining high-quality older adult care is essential, as excessive strain can impact both mental health and service quality. Automatic monitoring systems offer a promising solution; however, their real-world implementation requires a robust human activity recognition (HAR) framework that accounts for older adult–specific mobility and health variations. Existing HAR methods mostly use RGB or RGB-D camera [25] and inertial measurement units [26]. Wearable inertial measurement units, although effective, can be intrusive and uncomfortable. In contrast, depth sensors and RGB cameras provide a nonintrusive, cost-effective alternative, advancing HAR through wireless sensor networks and the Internet of Things.

HAR approaches typically rely on RGB-based [27-29] or skeleton-based [30-32] methods, which perform well in general settings but degrade significantly for older adult individuals due to posture and mobility differences influenced by health conditions. A key limitation of traditional HAR models is the lack of care assessment scores (CASs), which are critical in older adult care. Caregivers assign CASs such as the activities of daily living score, Barthel Index, and care level (CL) to assess mobility, physical dependency, and assistance needs. These scores provide vital insights into an individual’s functional abilities, yet state-of-the-art HAR methods fail to incorporate them, limiting their applicability to older adult populations. Since posture and motion patterns are directly affected by health conditions, neglecting these factors leads to poor generalization. Moreover, widely used HAR datasets such as RGBD-HuDaAct [33], 3D Action Pairs [34], MSR-DailyActivity [35], NTU RGB+D 120 [36], and UTD-MHAD [37] lack older adult–specific data, as they primarily feature younger or healthier participants. This demographic gap hinders HAR models from accurately recognizing older adult activities. To address this challenge, integrating CASs into HAR models is essential for personalized, context-aware monitoring, enabling more adaptive and accurate older adult activity recognition.

### Related Works

Traditional HAR methods struggle with older adult individuals, leading to research on tailored models and datasets. However, challenges remain. This section reviews state-of-the-art HAR and older adult HAR methods using skeleton and video data, together with older adult–specific datasets.

### Older Adult Activity Recognition

RGB-based HAR is widely used, particularly for monitoring applications. It primarily extracts motion information from video frames and can be classified into 2 categories: 2-stream networks and 3D convolutional networks. Two-stream networks leverage RGB data for spatial representation and optical flow for temporal dynamics [38-44]. However, computing optical flow is computationally expensive, creating bottlenecks in real-time applications. In contrast, 3D convolutional networks [45-48] aim to capture spatiotemporal features directly from video sequences but face challenges related to occlusions, camera motion, and environmental complexities [49]. These limitations are even more pronounced in older adult activity recognition, where subtle motion variations and fine-grained details play a crucial role. Skeleton-based approaches offer a compact and robust representation of human motion by focusing on skeletal joints and their temporal evolution. Early works used spatial graph-based models [50-53], while later studies introduced temporal relationships through recurrent and convolutional architectures [54-58]. However, skeleton-based methods lack environmental context, limiting their effectiveness in recognizing human-object interactions—an essential factor for assessing functional abilities in older adult individuals. To address the limitations of unimodal methods, researchers have explored multimodal fusion techniques that integrate RGB and skeleton data [59,60]. These methods typically extract features from each modality independently before performing fusion, with some incorporating contextual information such as human-object interactions and location data [61-63]. Despite improved recognition accuracy, existing multimodal approaches struggle with effective feature aggregation, as irrelevant modality-specific information can degrade overall performance. More importantly, these methods are predominantly trained on young, healthy individuals and lack adaptations tailored for older adult populations. A notable older adult HAR system was introduced in the study by Kim et al [64], using depth video and skeleton joint features. While effective in controlled environments, this approach failed to generalize well to real-world scenarios due to the exclusion of contextual and health-related mobility variations and the lack of real-world continuous activity patterns in the training data. Another older adult HAR method proposed a feature fusion model combining handcrafted and deep-learned features using a dedicated dataset [65]. However, this approach remains limited by its reliance on a homogeneous environment and its inability to capture long-range dependencies.

To overcome the limitations of existing methods, we propose a cross-modal and personalized HAR approach that considers older adult health conditions. Our base model is built on transformers, which have demonstrated exceptional performance in HAR due to their ability to model long-range dependencies through self-attention mechanisms. Most transformer-based HAR methods process RGB frames as input tokens [66,67] or, less commonly, skeleton data [68,69]. However, these approaches suffer from high computational costs, restricting their applicability in real-time older adult care environments. In addition, existing transformer-based models do not efficiently integrate cross-modal information, limiting their ability to leverage multimodal dependencies for older adult HAR. To address these challenges, we propose a depth map-based approach instead of RGB to reduce computational costs. We have designed a care-aware attention mechanism (CAM) and incorporated it into the spatial layer, replacing the standard self-attention mechanism in transformers. This effectively facilitates cross-modal analysis by integrating care assessment information, skeleton data, and depth images, thereby enhancing older adult HAR performance.

### Older Adult Dataset

Although most benchmark HAR datasets are designed for the general population, some datasets have been specifically collected to study older adult individuals. EGOFALLS [70] focuses on fall detection using egocentric camera data, containing 10,948 video samples from 14 participants, including 12 young adults and only 2 older adult individuals. Despite its large size, the dataset has a significantly small sample of older adult participants. Additionally, its controlled environment and lack of health condition–based variations make it unsuitable for real-world application training. The Toyota Smarthome Dataset [71] captures daily living activities using 7 Kinect sensors from 18 volunteers aged 60-80 years over 8 hours in a controlled apartment setting. While it includes interactions with household objects, its limitations stem from the controlled environment and the absence of health condition–based variations. IntelliRehabDS [72] was collected using a Kinect motion sensor and comprises 9 repetitive gestures performed by 29 individuals, including 15 patients and 14 healthy controls. It provides 3D body joint coordinates and depth maps, annotated for gesture type and position (sitting or standing). However, the dataset has a narrow older adult age range (20-60+ years), is collected in controlled conditions, and lacks health condition–specific data. ETRI-Activity3D [73] is a large-scale dataset that includes RGB videos, depth maps, and skeleton sequences from 100 participants—50 older adult individuals (aged 64-88 years, with an average age of 77 years) and 50 younger adults (average age of 23 years). Despite its scale, the dataset has limitations, including a lack of intervention data, health condition–based variations, and continuous activity recordings. Overall, existing older adult HAR datasets suffer from small and homogeneous older adult sample sizes, controlled environment constraints, and limited continuous activity data, often lacking health condition–specific variations. To address these issues, we collected our dataset in a real care facility without intervention or manipulation, capturing mealtime sessions of 28 older adult participants aged 62-95 years, representing 5 distinct CLs.

### Objective and Contributions

In this work, our objective is to develop an improved older adult activity recognition method that integrates care assessment information with motion data to effectively capture and understand movement variability caused by different health conditions. To achieve this, we incorporated CL, one of the most widely used CASs in older adult care facilities, due to its availability and relevance in evaluating functional abilities, and introduced a care-assessment-aware spatiotemporal transformer (CSTT) that adapts its attention to key points and depth-based motion patterns based on an individual’s CL, enabling personalized feature prioritization and improved activity prediction. We also addressed the issue of the lack of suitable data and collected a real-world older adult activity dataset incorporating CL information. The key contributions of our work can be summarized as follows:

1. We propose the first care-assessment-aware activity recognition approach by modeling the correlation between health conditions using CL and movement patterns to personalize and improve older adult activity recognition.
2. We propose CSTT, integrating skeleton, depth heatmaps, and CL information. Our proposed CAM dynamically adjusts focus based on care needs, ensuring personalized recognition while enhancing robustness and efficiency.
3. We present the first motion dataset with CL information, capturing real-world mealtime sessions to reflect aging and health impacts on mobility with high ecological validity.
4. This work improves older adult activity recognition by integrating care assessment information and capturing motion variations influenced by these conditions for improved accuracy.

## Methods

### Study Design

**Figure 1:**
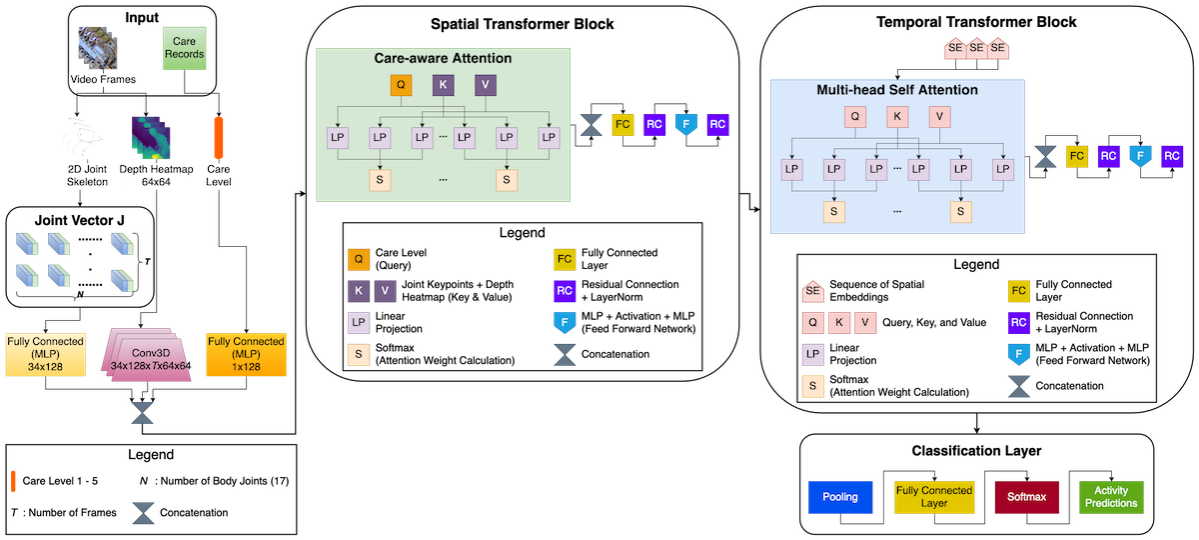
Care-assessment-aware spatiotemporal transformer architecture. This transformer enhances older adult activity recognition by integrating joint skeleton data, depth heatmaps, and CL information. The Spatial Transformer Block uses a care-aware attention mechanism, where the care level acts as a query to capture spatial relationships. The Temporal Transformer Block uses multihead self-attention to model temporal dependencies across frames. Finally, the Classification Layer processes the learned representations to predict activity labels using a fully connected network and softmax activation.

In this work, we tackled the challenge of older adult activity recognition, which is complicated by pose deformities and motion limitations in older adults. To address these issues, we proposed a heterogeneous spatiotemporal motion transformer with CAM, specifically designed for recognizing older adult activities. For model training, we collected a dataset with informed consent and ethical permission, 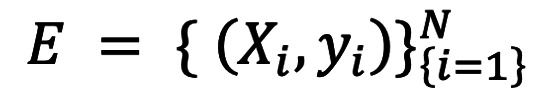 where, *X_i_* consists of body key points, depth images, and CL information, while *y_i_* represents the corresponding activity label, and *N* denotes the number of training samples. The objective is to learn a function *f*: *X* → *y* that accurately classifies activities. In this section, we provide a detailed explanation of our data processing, followed by an in-depth discussion of the CSTT transformer. The overall architecture is illustrated in Figure 1.

### Data Collection and Validation Method

#### Overview

**Figure 2:**
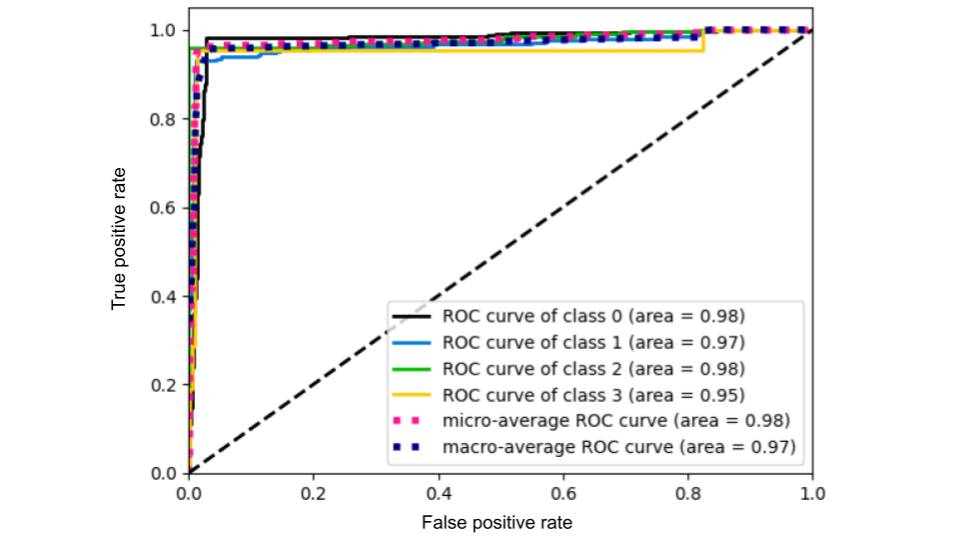
Model performance evaluation: the receiver operating characteristic curve illustrates care-assessment-aware spatiotemporal transformer’s prediction accuracy across different activities (class 0: sitting, class 1: eating, class 2: stand up, and class 3: trying to stand up). ROC: receiver operating characteristic curve.

The dataset was gathered in partnership with Global Care, a care facility in Japan, dedicated to supporting patients with dementia, which is equipped with an in-house video-monitoring system. The camera used for the monitoring is the AXIS M3048-P [74], a cost-effective fixed dome fish-eye camera featuring a 12-megapixel sensor. This camera is designed to provide a comprehensive 360° view of the surroundings, with distortion-corrected display options such as panoramic views, specific areas, corridors, corners, and quad displays, all offering exceptional sharpness. Additionally, the camera comes prefocused, eliminating the need for manual adjustments. To maintain the authenticity of the care environment, we chose not to incorporate any additional sensors. In collaboration with the care facilities, we accessed their video recordings, which specifically captured activities of 51 older adult people. For dataset 1, data was collected from 28 elderly during lunch mealtime from 3 different sites over a span of 15 days. Each recorded session lasted between 30 and 60 minutes. Older adult participants’ ages are ranging from 64 to 95 (mean 79.5) years. The placement of each participant was predetermined and managed by the staff of the care facility. To maintain the facility’s natural workflow, our team provided no instructions or interventions. In site 2, as shown in Figure 2, patient 7 was assigned multiple positions, as their location was occasionally adjusted by the staff during certain sessions. This variation in positioning was carefully considered during the data processing phase to ensure the accuracy of the labels. For dataset 2, the other 23 participants’ data were collected from open-gathering space of dining and living area in site 4 from 2 different floor. Participants are aged between 71 and 92 (mean 81.5) years. The data were collected over 15 days period through continuous 24 hours monitoring. The site layouts are given in Multimedia Appendix 1.

Due to the limitations in posture and mobility, the activities of older adult individuals differ significantly from those of healthy individuals. Similar activities may present with different postures depending on CL, while different activities might appear with similar postures across different CL groups. As a result, CL may infer crucial insights for older adult activity recognition. To address this, we have collected each older adult person’s CL alongside the video data. The score was provided by the medical professionals based on assistance requirements in meal, bath, excretion, movement, and dress-up. These levels, ranging from 1 (minimal assistance) to 5 (maximum assistance), determine the extent of supervision and support required [75]. Table 1 shows the explanation regarding the CL. For easier understanding of the readers, we have categorized the CLs based on assistance requirements into low, mid, and high.

From the recorded videos, skeleton data and depth images were extracted for analysis. For the purpose of this study, only skeleton data, depth images, and care-related information were used, while RGB images were excluded from the analysis. In Table 2, baseline comparison is shown between the 2 collected dataset.

Comparing the components between the 2 datasets in Table 2, we found that gender, care assistance requirement, and data collection time are significantly different. The age does not have much variation because the target group of our research is 60 above to 100.

To highlight the necessity of incorporating care context with motion data for activity recognition, we used 2 similarity comparison approaches. The first involves calculating pairwise similarity among different CL groups, while the second focuses on comparing the pairwise similarity between low and medium CL groups for various activities. To compute similarity scores, we used mean per joint angle difference (MPJAD), cosine similarity (CS), and histogram of oriented gradients (HOG) similarity. Detailed explanations of the score calculation methods are given in the following sections:

**Table 1:** Care-level interpretation for the older adult individuals.

AR^a^	CLs^b^	Older adult condition
Low	1 and 2	Older adult individuals in this group can move independently or with minimal aid. They sometimes require monitoring and reminders for meals, excretion, and baths. For dress-up, they sometimes need monitoring or partial assistance. They generally maintain an upright posture, with only minor stooping due to age-related spinal degeneration.
Mid	3	Older adult individuals at this stage require full support from caregivers for movement; however, they can stand up with the support of assistive devices. They require monitoring, reminders, and partial assistance for meals, excretion, and baths. For dress-up, they need full assistance. Posture is often characterized by forward leaning or hunching due to weakened core muscles and joint instability. Sitting may involve slumping as maintaining an upright position becomes difficult.
High	4 and 5	Older adult individuals at this stage require full support from caregivers for everything. They are predominantly immobile. Postural control is severely compromised. No standard posture can be seen.

^a^AR: Assistance requirement.

^b^CLs: care levels.

**Table 2:** Baseline comparison between the datasets.

Components		Dataset 1	Dataset 2	*P* value
Age (years), mean (SD)		79.5 (8.95)	81.5 (6.06)	.35
**Gender**				
	Men	10	20	<.001
	Women	18	3	
**Care assistance requirement**				
	High	6	1	<.01
	Medium	8	18	
	Low	14	4	
Data collection time (minute)		750	21,600	<.001

#### Mean per Joint Angle Difference

MPJAD measures the average angular difference between 2 motion sequences across all frames and joints. Given 2 motion sequences and , the MPJAD is computed as:



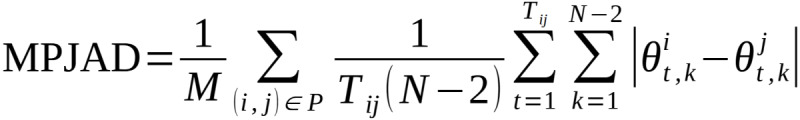



(1)

Where *P* is the set of all motion sequence pairs, *M* is the number of motion sequence pairs, *T_ij_* is the number of frames common between sequences *S_i_* and *S_j_*, *N* is the total number of joints (17 in this case), 
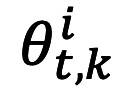
 is the joint angle at joint *k* and frame *t* in sequence *S_i_*, and 
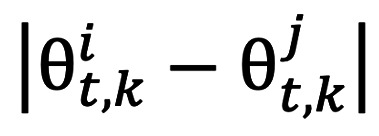
 is the absolute difference in joint angles. A lower MPJAD value indicates a higher similarity between motion sequences. The 2 motions follow similar joint movement patterns. A higher MPJAD value indicates greater dissimilarity between motion sequences. The 2 motions have significantly different joint movements.

#### Cosine Similarity

CS measures the angle between 2 vectors in a multidimensional space. Given 2 motion sequences *S_i_* and *S_j_*, the CS is computed by following the steps:

Derive the cosine dissimilarity (used to measure homogeneity):



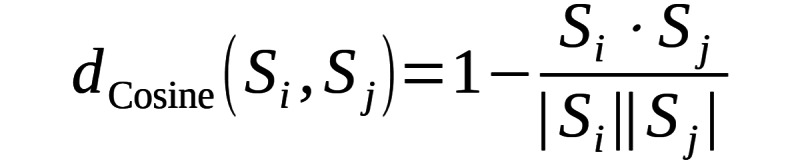



(2)

Where *S_i_* and *S_j_* are the flattened motion sequences represented as vectors. *S_i_* · *S_j_* is the dot product of the 2 vectors. |*S_i_*| and |*S_j_*| are the Euclidean norms (magnitudes). Compute the mean pairwise cosine dissimilarity within the class:



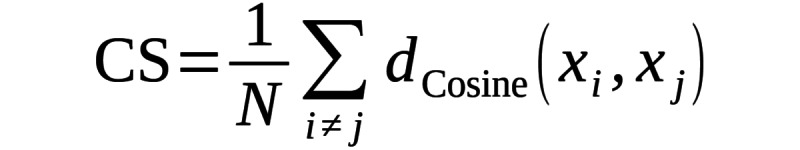



(3)

Where *N* is the total number of unique pairs (*i,j*) within the class. Lower values of cosine dissimilarity indicate higher homogeneity (motion vectors within the class are closely aligned). Higher values of cosine dissimilarity indicate lower homogeneity (motion vectors within the class are more divergent).

#### HOG Similarity

HOG measures how structurally similar 2 images are based on their gradient information. Once the HOG feature vectors *H_1_* and *H_2_* are extracted for 2 images, their similarity is computed using the CS formula:



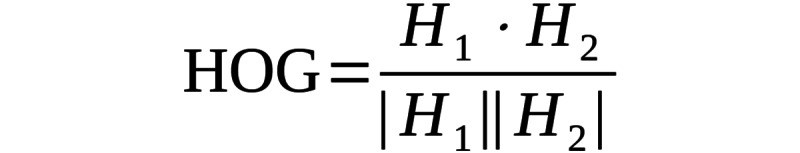



(4)

Where *H_1_* · *H_2_* is the dot product of the 2 HOG feature vectors. |*H_1_*| and |*H_2_*| are the Euclidean norms of the vectors. If the HOG score is 1, it means the images have nearly identical edge and gradient structures, and if it is 0, it means the images have no similarity in gradient structure.

### Ethical Considerations

This work involved human participants or animals in its research. Approval of all ethical and experimental procedures and protocols was granted by the research ethics board at Kyushu Institute of Technology (application no. 24-15). No participant compensation was provided. The data are not publicly available but they would be shared with upon the collaboration request only for research purpose to the applicants who adhere to the ethical and privacy policy of the ethical committee after careful consideration.

### Skeleton Data Generation

To ensure robustness, we used skeleton data in our study. To get the skeleton data we used YOLOv7 [76], a state-of-the-art real-time object detection framework that builds upon the foundational YOLO (You Only Look Once) [77] family, offering enhanced performance in terms of accuracy. It maintains high precision in both detection and key point localization, and the architecture can handle multiple scales and dense environments effectively. The input is an RGB image, *I*_RGB_∈R*^H^*^×^*^W^*^×3^ where *H* is the height of the image, *W* is the width of the image, and 3 represents the number of color channels (ie, red, green, and blue). YOLOv7 identifies bounding boxes for human figures in the input RGB image. Each bounding box *B_k_* is represented as *B_k_* = (*x*_min_,*y*_min_,*x*_max_,*y*_max_,*c_k_*), where (*x*_min_,*y*_min_) and (*x*_max_,*y*_max_) define the corners of the box and *c_k_* is the confidence score. Within each bounding box, the model then predicts the locations and confidence scores for the 17 key points *X* = {*X*_1_,*X*_2_,….,*X*_17_}, where each key point *X_i_* is defined as *X_i_* = (*x_i_*,*y_i_*,*c_i_*), i∈{1,2,…,17}. Here, *x_i_*,*y_i_*: 2D coordinates of the *i*th key point in the image and *c_i_*: confidence score indicating the likelihood that the *i*th key point is correctly detected (*c_i_* ∈ [2]). So, the whole body can be represented as:



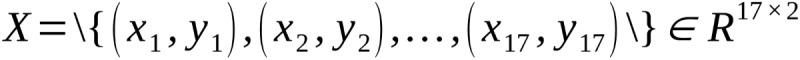



(5)

where the total key point feature vector has 34 dimensions (17 key points × 2 coordinates).

### Depth Image Generation

Skeleton data are highly robust but fail to capture the environmental context. In contrast, RGB data often include excessive information, which can lead to confusion due to varying lighting conditions. However, depth images offer valuable spatial insights, providing detailed information about the distance and positions of objects and body parts. Therefore, we generated depth maps from RGB images and incorporated them into our study. To achieve this, we used the “Depth Anything” model [78], an advanced monocular depth estimation technique that transforms RGB images into depth maps. This model leverages deep learning (DL) to predict pixelwise depth values based on visual features in the image. Trained on extensive datasets of RGB-depth pairs, the model knows how to estimate depth using visual information alone. Each pixel in the image has 3 values corresponding to its red, green, and blue intensities. The goal of the model is to convert this 3-channel image into a depth map, where each pixel value corresponds to the distance from the camera to the object in the scene. Let *f*_θ_ represent the trained model that maps an RGB image IRGB to its predicted depth map *D*. The model was learned from large datasets containing paired RGB images and their corresponding depth maps during training. The depth estimation function is given by *D* = *f*_θ_(*I*_RGB_). Then, each pixel of the depth map represents that the estimated distance to the camera can be denoted by



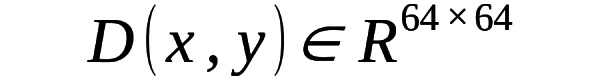



(6)

where (*x, y*) are the coordinates of the pixel in the image. The value *D* (*x, y*) represents the depth of the corresponding point in the scene.

### Care-Assessment-Aware Spatiotemporal Transformer

Our proposed CSTT analyzes body key points, heatmaps, and CL to predict human activities in a personalized manner. As a spatiotemporal heterogeneous transformer, it captures spatial features (key points and heatmaps) and temporal dynamics (motion sequences over time) while integrating multiple modalities with distinct representations—numerical key points, image-based heatmaps, and scalar CL—through CAM. In the Spatial Transformer, key points and heatmaps are processed to extract movement-related features, with the CL serving as an attention guide, determining which body movements and heatmap regions are most relevant. This enables the model to dynamically prioritize motion patterns based on an individual’s care needs. The Temporal Transformer then analyzes the sequence of spatial features, capturing motion dynamics over time. Learning temporal dependencies helps recognize activities and transitions between postures. Finally, the extracted spatiotemporal features are passed to a classifier, which predicts the activity category.

#### Spatial Transformer

The Spatial Transformer extracts meaningful representations from the key points and heatmap while considering the CL as a crucial guiding factor. The CL attends to the key points and heatmap, allowing the model to emphasize relevant body parts or movements based on the older adult person’s health condition.

#### Feature Embedding

##### Overview

To transform the raw input data into a meaningful and structured format suitable for further processing, we used an embedding mechanism. This step is crucial as it converts different types of input data—body key points, heatmaps, and CL—into a shared latent representation that can be effectively used by the transformer model. By mapping these diverse inputs into a common latent space of dimension *d*, the embedding process ensures that the model can seamlessly integrate and compare information from multiple modalities, facilitating efficient learning and cross-modal interactions. There are 3 types of feature embedding done here as mentioned in the following sections.

##### Key Point Embedding

The 2D pose key points (17 joints, each with (x,y) coordinates) are flattened and passed through a linear layer:



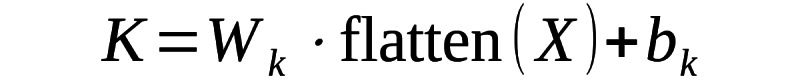



(7)

Where *X* ∈ R^B×17×2^ is the input key point tensor and *W_k_* ∈ R^(17×2)×^*^d^* is the weight matrix, which learns spatial dependencies. *K* ∈ R*^B^*^×^*^d^* is the key point embedding.

##### Heatmap Embedding

The heatmap (grayscale image of size 64 × 64) is processed through a 3D convolution layer to capture local spatial dependencies and activation patterns:



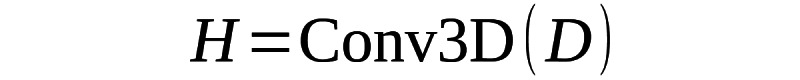



(8)

where, *D* ∈ R*^B^*^×1×64×64^ is the heatmap input. *H* ∈ R*^B^*^×^*^T^*^×^*^d^* is the heatmap embedding after convolution, where *T* represents the number of spatial patches.

##### CL Embedding

The CL (a scalar value) is passed through a linear layer:



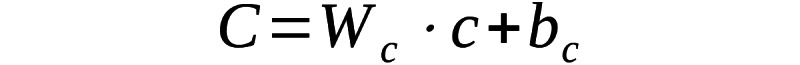



(9)

Where *c* ∈ R*^B^*^×1^ is the CL tensor and *Wc* ∈ R^1×^*^d^* is the weight matrix. *C* ∈ R*^B^*^×^*^d^* is the CL embedding.

#### Care-Aware Attention Mechanism

##### Overview

Unlike conventional transformer self-attention mechanisms that treat all inputs equally, our proposed CAM dynamically adjusts attention based on CL. Using CL embeddings as queries in a multihead attention mechanism prioritizes key body parts and movements. Key points and heatmaps form key value pairs, allowing the model to focus on relevant features. The attended features are refined through a feedforward network, enhancing learning. By explicitly integrating CL into feature learning, CAM improves interpretability and efficiency, making activity recognition more personalized and accurate. The entire process is depicted as follows:

##### Attention Score Computation

The traditional attention mechanism follows the scaled dot product attention formulation:



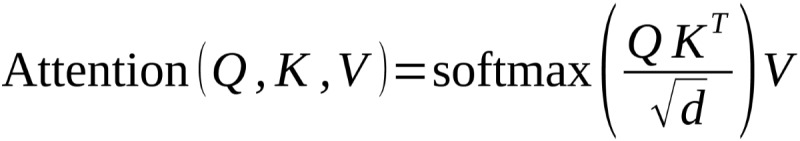



(10)

Here, we made the modification in the embeddings of *Q*, *K*, and *V* values. CL embedding *C* ∈ R*^B^*^×1×^*^d^* is assigned to *Q* (Query). Concatenated Key point + Heatmap Features [*K*,*H*] ∈ R*^B^*^×(^*^T^*^+1)×^*^d^* is assigned to both *K* (Key) and *V* (Value). So the modified attention weights computation formula is:



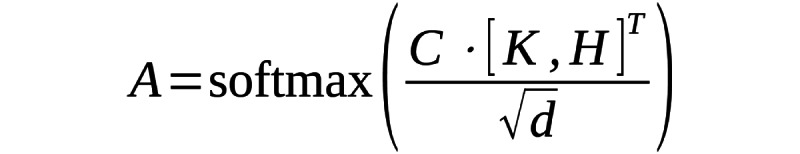



(11)

This results in a weighted sum of key points and heatmap features, emphasizing relevant information based on the CL.

##### Multihead Attention

To enhance model expressiveness, we use multihead attention, where different heads capture different aspects of the input:



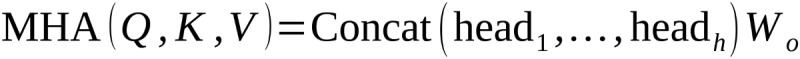



(12)

where each head performs attention independently, and the outputs are concatenated and projected using *W_o_*.

##### Feedforward Network

After attention, the output is passed through a feedforward network:



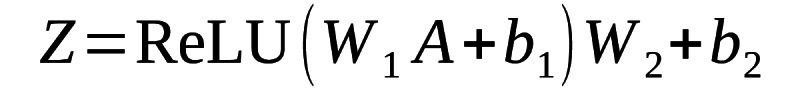



(13)

This enhances the feature representation before passing it to the temporal transformer.

#### Temporal Transformer

The temporal transformer is designed to capture and model the sequential dependencies that exist across frames. Since traditional transformers do not inherently account for the order of sequences, we incorporate positional encoding to inject information about the temporal order of the frames, allowing the model to distinguish between the different points in time:



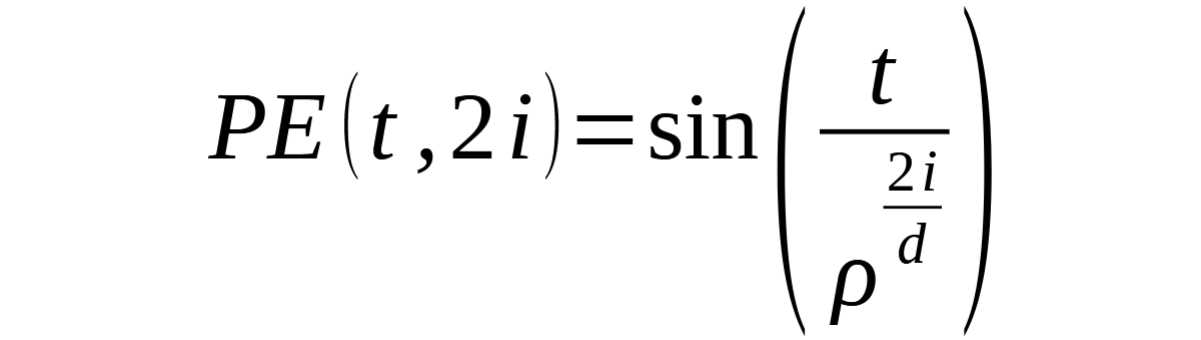



(14)

Where ρ is a constant eprecally calculated and the value of ρ is 10,000. The Transformer Encoder then applies self-attention across the temporal dimension, processing a sequence of spatial features *Z_t_* obtained from various timesteps. This process is mathematically represented as:



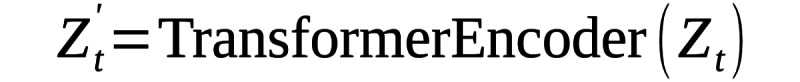



(15)

Where *Z_t_* ∈ R*^T^*^×^*^d^* is the temporal sequence of spatial representations and 
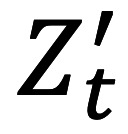
 is the transformed sequence after passing through the encoder. Throughout this process, the model allows the frames to “attend” to one another, meaning each frame is evaluated in relation to others. This enables the model to capture the dependencies of motion, such as recognizing how a body part moves or changes over time, effectively tracking the progression of movement across frames. After the temporal sequence is processed, the features are aggregated using mean pooling to summarize the sequence of attended frames into a single fixed-size representation:



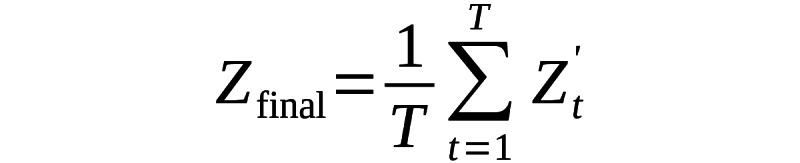



(16)

This pooled feature vector encapsulates the entire movement sequence, providing a compact yet informative summary. Finally, this aggregated representation is passed into a classifier for further interpretation, enabling the model to make predictions based on the captured temporal dynamics.

#### Classification

The final feature vector encapsulates the entire activity, providing a contextual representation of the movement. After the pooled feature vector is obtained from the Temporal Transformer, it is passed through a softmax classifier for classification:



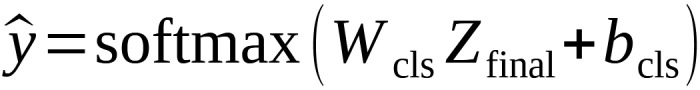



(17)

In this equation, *W*_cis_ and *b*_cis_ are learnable parameters that the model optimizes during training. Specifically, *W*_cis_ is responsible for projecting the final feature vector into activity categories, effectively mapping the feature space to the set of possible classes. The softmax function then assigns probabilities to each of these activity categories, indicating the likelihood of each class being the correct one.

## Results

In this work, we have proposed a care-assessment-aware approach for older adult activity recognition. This section presents the validation results for our dataset and evaluates the model’s performance. In addition, we have conducted an ablation study and provided a detailed discussion of the obtained results.

### Dataset Validation

To assess the dataset and demonstrate the importance of integrating care context, we applied 2 similarity-based evaluation strategies. The first measures pairwise similarity across all care-level groups, shown in Table 3 and the second examines pairwise similarity specifically between the low- and medium-care groups for each activity, shown in Table 4. Similarity was quantified using MPJAD, CS, and HOG similarity.

From Table 3, it is evident that the movement patterns of eating and sitting activities in the high care assistance group differ significantly from those in the other 2 groups. Since individuals in this category are almost immobile, activities such as trying to stand up and standing up are absent. For the low and medium care assistance groups, we conducted a pairwise similarity analysis of activities, as we observed that the motion patterns of trying to stand up and standing up share some similarities and yet exhibit notable differences. From Table 4, we can see that, despite being different activities, certain motion patterns show a high degree of similarity. In particular, the pairs eating–trying to stand up and standing up–trying to stand up demonstrate strong similarities, which is uncommon in standard activity recognition scenarios. These findings confirm that care context plays a crucial role in older adult activity motion patterns. To strengthen our claim, we also analyzed motion patterns using a comparative group of young adults. Since the study primarily focuses on older adult participants, these additional results are provided in Multimedia Appendix 1 for reference. All these comparison results validate our claim that care information plays a significant role in accurately distinguishing activities.

**Table 3:** Pairwise similarity calculation among different care level groups.

	Mean per joint angle difference			Cosine similarity				Histogram of oriented gradients similarity		
	^a^L-H	^b^H-M	M-L^c^	L-H	H-M	M-L	L-H		H-M	M-L
E^d^	0.95	0.92	0.18	0.95	0.92	0.16	0.07		0.14	0.91
S^e^	0.95	0.92	0.24	0.95	0.92	0.27	0.07		0.14	0.87
T^f^	N/A^g^	N/A	0.49	N/A	N/A	0.51	N/A		N/A	0.25
SU^h^	N/A	N/A	0.78	N/A	N/A	0.80	N/A		N/A	0.34

^a^L-H: low and high care–level pair.

^b^H-M: high and medium care–level pair.

^c^M-L: medium and low care–level pair.

^d^E: eating.

^e^S: sitting.

^f^T: trying to stand up.

^g^N/A: not applicable.

^h^SU: stand up.

**Table 4:** Activity pairwise similarity calculation between low and medium care–level groups. The first activity is from the medium group.

Activity pair	Mean per joint angle difference	Cosine similarity	Histogram of oriented gradients similarity
E^a^-S^b^	0.91	0.93	0.14
E-T^c^	0.21	0.23	0.91
E-SU^d^	0.56	0.60	0.72
S-E	0.92	0.95	0.12
S-T	0.55	0.59	0.71
S-SU	0.54	0.59	0.72
T-E	0.22	0.24	0.90
T-S	0.56	0.61	0.72
T-SU	0.21	0.23	0.88
SU-E	0.56	0.59	0.73
SU-S	0.51	0.53	0.71
SU-T	0.25	0.27	0.89

^a^E: eating.

^b^S: sitting.

^c^T: trying to stand up.

^d^SU: stand up.

### Model Performance Evaluation

**Figure 3:**
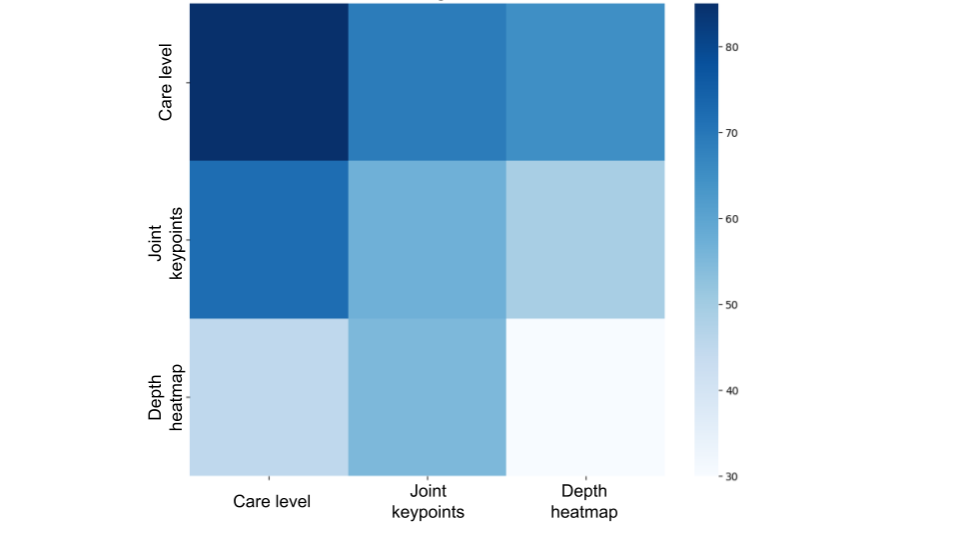
Model performance evaluation: attention weight matrix demonstrates the effective incorporation of care level through care-aware attention mechanism in care-assessment-aware spatiotemporal transformer.

**Figure 4:**
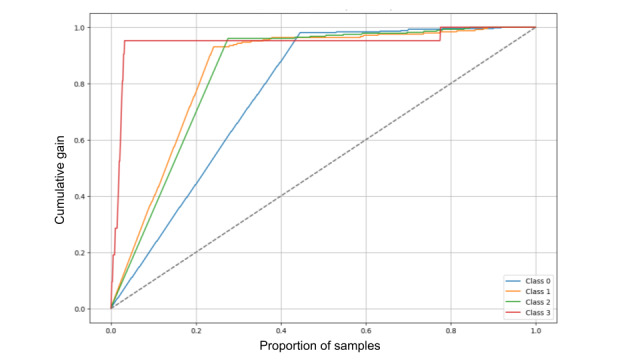
Model performance evaluation: the cumulative gain plot (one vs all) illustrates care-assessment-aware spatiotemporal transformer’s prediction accuracy across different activities (class 0: sitting, class 1: eating, class 2: stand up, and class 3: trying to stand up). Here, red, orange, green, blue, and dotted lines represent class 3, class 1, class 2, class 0, and baseline accordingly.

To evaluate the model’s performance, we analyzed the attention weight matrix (AWM), the receiver operating characteristic curve, and the cumulative gain plot. The AWM in Figure 3 validates that the CSTT model efficiently integrates CL, body key points, and depth heatmaps through CAM. The model dynamically adjusts the importance assigned to different input features, demonstrating its ability to capture hierarchical dependencies. Notably, CL serves as a guiding factor, influencing how attention is distributed across other features. The receiver operating characteristic curve in Figure 2 evaluates the model’s ability to distinguish activity classes by plotting the true-positive rate versus the false-positive rate. The area under the curve (AUC) values range from 0.95 to 0.98, indicating excellent classification performance. The microaverage AUC (0.98) reflects strong overall accuracy, while the macroaverage AUC (0.97) shows balanced performance across classes. Classes 0, 1, and 2 have the highest AUC values, ensuring clear separation, while class 3 (0.95) shows slight overlap but still performs well. The cumulative gain plot in Figure 4 highlights the model’s ability to rank correct predictions early. The steep rise in curves shows effective prioritization. This confirms that the CSTT efficiently ranks true positives early, which is critical for applications needing confident and rapid classification.

We selected both machine learning and DL models as baselines to evaluate our approach. Table 5 presents the results with and without CL information, except for our model, where care context is essential when the data are benchmarked using a cross-day approach, applying an 80-20 train-test split. Machine learning models used only skeleton data, resulting in comparatively lower performance. However, adding just care context significantly improved all models’ performance. Our model outperformed the traditional spatiotemporal transformer, achieving a 5% higher accuracy and a 9% increased *F*_1_-score.

**Table 5:** Comparison with baseline methods.

Method	Without care (C) level					With care (C) level			
	P^a^	R^b^	*F*_1_-score	A^c^	P		R	*F*_1_-score	A
Random Forest (S^d^)	0.58	0.65	0.56	0.65	0.69		0.69	0.69	0.69
XG Boost (S)	0.67	0.69	0.66	0.69	0.74		0.74	0.74	0.74
CNN^e^ (S + D^f^)	0.76	0.75	0.76	0.77	0.82		0.82	0.81	0.80
ResNet (S + D)	0.80	0.74	0.76	0.86	0.85		0.85	0.85	0.85
TSTT^g^ (S + D)	0.82	0.83	0.82	0.83	0.83		0.91	0.87	0.91
Our model	N/A^h^	N/A	N/A	N/A	*0.97* ^i^		*0.96* ^i^	*0.96* ^i^	*0.96* ^i^

^a^P: precision.

^b^R: recall.

^c^A: accuracy.

^d^S: skeleton data.

^e^CNN: convolutional neural network.

^f^D: depth image.

^g^TSTT: traditional spatiotemporal transformer.

^h^N/A: not applicable.

^i^Best performance results values are in italics to highlight the importance.

### Ablation Study

In the ablation study, we removed components from the key and value pairs to analyze their impact. Also, we have randomly removed some key points to create partial skeleton data. We compared the traditional spatiotemporal transformer without the CAM layer, a care-assessment-aware transformer using only skeleton data (CSTT [S + C]), a care-assessment-aware transformer using only partial skeleton data (CSTT [Sp + C]), one using only depth images (CSTT [D + C]), our full model integrating partial skeleton data (CSTT [Sp + D + C]), and our full model integrating all (CSTT [S + D + C]). As shown in Table 6, CSTT (S + C) outperforms CSTT (D + C), aligning with the AWM analysis in Figure 3. Also, CSTT (Sp + C) performs poorly compared with others, but CSTT (Sp + D + C) performance is in the acceptable range. This shows that the model is comparatively robust.

**Table 6:** Ablation study comparison.

Method	P^a^	R^b^	*F*_1_-score	A^c^
TSTT^d^ (S^e^ + D^f^)	0.82	0.83	0.82	0.83
TSTT (S + D + C^g^)	0.83	0.91	0.87	0.91
CSTT^h^ (S + C)	0.94	0.84	0.88	0.93
CSTT (Sp^i^ + C)	0.81	0.88	0.84	0.85
CSTT (D + C)	0.91	0.82	0.84	0.90
CSTT (Sp + D + C)	0.92	0.90	0.91	0.91
Our model	*0.97* ^j^	*0.96* ^j^	*0.96* ^j^	*0.96* ^j^

^a^P: precision.

^b^R: recall.

^c^A: accuracy.

^d^TSTT: traditional spatiotemporal transformer.

^e^S: skeleton data.

^f^D: depth image.

^g^C: care context.

^h^CSTT: care-assessment-aware spatiotemporal transformer.

^i^Sp: partial skeleton data.

^j^Best performance results values are in italics to highlight the importance.

### Deployment

All experiments were implemented using the PyTorch DL framework and executed on an NVIDIA RTX 4080 GPU. The models were trained with a batch size of 8, an initial learning rate of 0.001, a weight decay of 0.0005, and for a total of 25 epochs. The trained model achieved an inference speed of 0.0043 seconds per sample, with approximately 1.2 M parameters and 1.09 Giga Floating-Point Operations per Second, indicating high computational efficiency.

## Discussion

### Principal Findings

This study demonstrated that older adult activity recognition can be significantly improved by integrating care-level information into model design. The proposed CSTT effectively captured variations in motion patterns caused by differing health conditions and care requirements. Results revealed that even for the same activity, movements differed across CLs, while visually similar motions appeared in distinct activities (eg, eating vs trying to stand up). Despite class imbalance and naturally occurring variations, CSTT achieved high recognition performance. Misclassifications—such as confusion between eating and sitting—were primarily linked to overlapping movements during caregiver assistance. These findings validate that incorporating care assessment information enables more robust, context-aware, and personalized activity recognition, aligning closely with real-world monitoring needs in long-term older adult care environments.

### Care-Assessment-Aware Older Adult Activity Recognition

Older adult activity recognition presents unique challenges due to the pose deformities, motion limitations, and variations in mobility caused by differing health conditions. Traditional HAR models often fail to capture these subtleties, as they are designed primarily for younger, healthy individuals and do not incorporate health-related variations. Furthermore, existing HAR approaches used for monitoring predominantly rely on RGB-based methods or, to a lesser extent, skeleton data, both of which struggle with computational efficiency and real-time applicability in older adult care environments. Our goal is to develop a context-aware HAR model that integrates CL assessments with motion data for personalized and accurate older adult activity recognition. To achieve this, we introduced CSTT, a heterogeneous spatiotemporal motion transformer incorporating skeleton data, depth-based heatmaps, and CL information, with CAM for improved adaptability. To ensure that the model captures real older adult activity patterns, we collected a motion dataset with CL annotations, recording uninterrupted mealtime sessions in a care facility for greater ecological validity. For model training, we focused on 4 key activities: eating, sitting, trying to stand up, and standing up. These were selected based on real nursing challenges—monitoring whether individuals are eating or sitting idle (requiring intervention if necessary) and ensuring continuous supervision during standing attempts to prevent falls. Unlike controlled datasets, our activity selection reflects real-world monitoring needs, capturing long-range dependencies in continuous time data, which aligns more closely with practical older adult care scenarios.

### Performance of Care-Assessment-Aware Older Adult Activity Recognition

**Figure 5:**
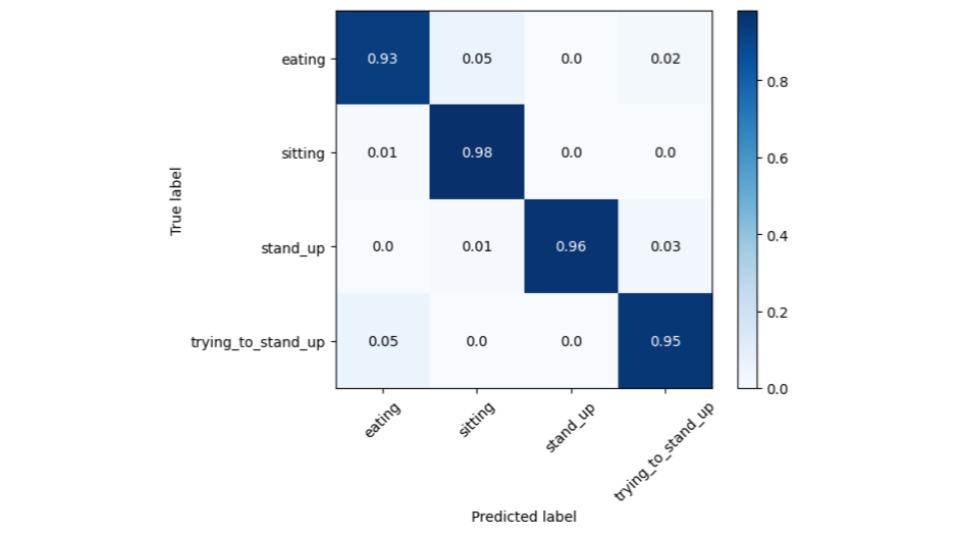
Normalized confusion matrix for all groups together.

**Figure 6:**
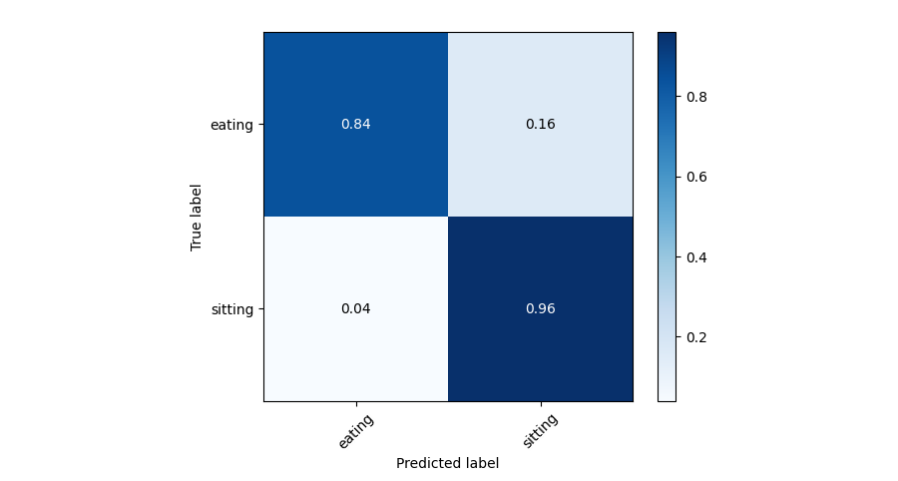
Normalized confusion matrix for high care assistance required group.

**Figure 7:**
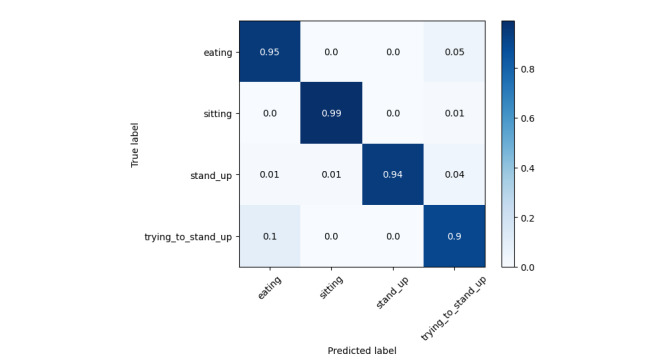
Normalized confusion matrix for medium care assistance required group.

**Figure 8:**
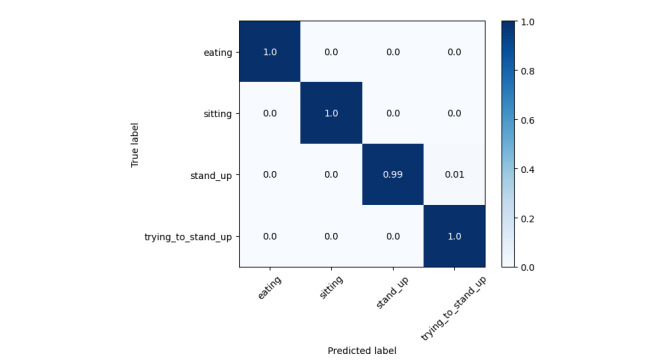
Normalized confusion matrix for low care assistance required group.

The results in Tables 3 and 4 support our initial hypothesis that older adult activity recognition differs from conventional methods, as their motion patterns are heavily influenced by their health conditions. Even for the same activity, movement can vary significantly across different CLs, while similar motion patterns may appear in completely different activities (eg, eating and trying to stand up). Our data collection was conducted without intervention to preserve real-world conditions. However, the dataset is highly imbalanced, particularly for the “trying to stand up” activity, which has significantly fewer samples than the other 3 classes. This reflects real-world scenarios but presents a challenge for recognition models. From Figure 5, the prediction accuracy for this class is 0.95, which is slightly lower than others. Among all classes, “eating” has the lowest accuracy at 0.93. Examining the confusion matrices provides insights into this misclassification. In Figure 6, eating is frequently confused with sitting, which is reasonable since caregivers often assist older adult individuals, reducing distinct motion patterns between these activities. In Figure 7, eating is often mistaken for trying to stand up, likely because older adult individuals extend their hands and lean forward for support when getting up—movements similar to hovering over a tray to pick up food. However, as seen in Figure 8, low care assistance individuals do not exhibit such overlapping movements, leading to perfect recognition of the eating activity. Based on overall performance, our model successfully outperforms the baselines.

### Current Limitations and Future Work

Although our dataset reflects real-world scenarios, it remains imbalanced, with activities such as “trying to stand up” having fewer samples. This affects classification accuracy (Figure 5), where “trying to stand up” achieves 0.95 and “eating” the lowest at 0.93 due to motion similarities, especially with caregiver assistance. We did not apply any data imbalance handling techniques, as “trying to stand up” naturally occurs less frequently in real-world settings. We did not apply data augmentation to address class imbalance because in older adult care monitoring, maintaining natural motion patterns and ecological validity is crucial. In cases of severe imbalance, augmentation risks producing unrealistic or biased samples, distorting class distributions, and leading to overfitting to synthetic data. The absence of CL annotations in existing datasets restricted direct comparisons with previous older adult HAR approaches. Although we manually annotated CLs for the ETRI Activity3D and Toyota Smarthome datasets to enable comparison, the potential inaccuracy of these inferred labels and the no representation of high-assistance older adult groups led us to include these results in Multimedia Appendix 2 rather than in the main manuscript. Future work will focus on annotating CLs in public datasets with medical professionals for comparative analysis. Due to ethical restrictions, our dataset cannot be publicly shared but may be accessed through collaboration. To support replication, we provide references and guidelines for collecting similar datasets with integrated CL information. Since our model depends on accurate CL assessments, misclassification can impact recognition, emphasizing the importance of expert collaboration in dataset development.

### Conclusions

In this work, we address the challenge of older adult activity recognition by considering the variability in movements influenced by CLs, which is often overlooked in existing datasets. To overcome this, we introduced a novel older adult motion dataset that includes CL information, collected from 51 older adult participants during real-world mealtime sessions in an ethical and privacy-preserving manner. We proposed CSTT, a spatiotemporal heterogeneous transformer model that integrates body key points, heatmaps, and CLs to predict activities in a personalized way by dynamically adjusting attention based on CLs. Our model surpassed the conventional spatiotemporal transformer, achieving a 5% higher accuracy and a 9% improvement in *F*_1_-score, highlighting the significance of incorporating CL data. However, limitations include dataset imbalance and the inability to compare with similar works due to a lack of comparable datasets, as well as ethical restrictions on sharing the dataset. In conclusion, our work lays the foundation for more accurate, context-aware older adult activity recognition, with future research focusing on dataset expansion, model refinement, and real-world applications in care settings.

## Appendix

Multimedia Appendix 1 Validation of care aware data collection and dataset reproducibility resources.

Multimedia Appendix 2 Generalization of care-assessment-aware spatiotemporal transformer by comparing performance on other data sets.

## Abbreviations

AUC: area under the curve
AWM: attention weight matrix
CAM: care-aware attention mechanism
CAS: care assessment score
CL: care level
CS: cosine similarity
CSTT: care-assessment-aware spatiotemporal transformer
DL: deep learning
HAR: human activity recognition
HOG: histogram of oriented gradients
LTC: long-term care
MPJAD: mean per joint angle difference
YOLO: You Only Look Once

## Data Availability

The data are not publicly available but they would be shared with upon the collaboration request only for research purpose to the applicants who adhere to the ethical and privacy policy of the ethical committee after careful consideration. Also, we have provided resources for reproducibility in Multimedia Appendix 1.
