# Supplementary material for: Integrating Care Context With Skeleton and Depth Information for Older Adult Activity Recognition in a Care Facility Using Care-Assessment-Aware Spatiotemporal Transformer: Method and Validation Study
Source: JMIR Aging. 2026 Apr 2;9:e80102. doi: 10.2196/80102 (PMC13087558; doi:10.2196/80102)
Supplement: Multimedia Appendix 1 [file aging_v9i1e80102_app1.docx]

## Multimedia Appendix 1

## Validation of Care Aware Data Collection:

Table S1. Pair-wise similarity calculation among different care level groups and young adults. Here, L-H: Low and High care level pair, H-M: High and Medium care level pair, M-L: Medium and Low care level pair; L-Y: Low care level and Young adult pair; M-Y: Midium care level and Young adult pair; H-Y: High care level and Young adult pair; E: Eating, S: Sitting, T: Trying to stand up, and SU: Stand up. The Young adult activity images were collected using the similar setup with the volunteers as subjects.


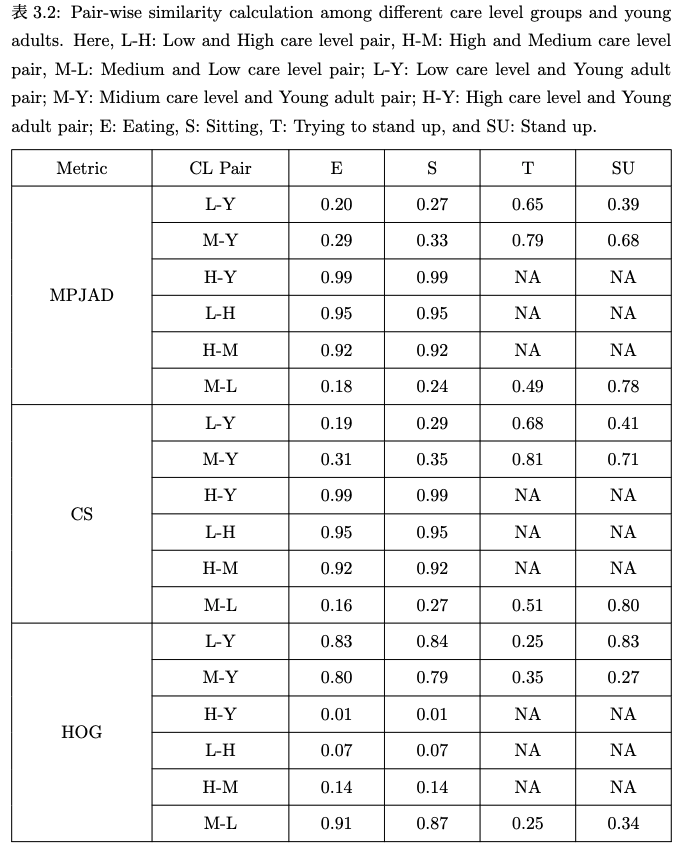


*** Similarity between high care level group with others is extremely low. For L and M have moderate similarity with Y in basic actions (E & S) but the gap widens considerably for transitional activities (T & SU), highlighting the progression of motor impairment and altered biomechanics with increased care needs. This validates our calim further for care information integrated datasets.

## Dataset Reproducibility:

Eventhough we are unable to disclose elderly identifying information we are sharing the data collection layout and care level scoring to make the study reproducible for data set 1. Table2: Subject Scoring Used in This Study. Here, Independence: No kind of assistance is required; Check: Nurses need to check elderly if they completed the task of not; Reminder: Nurses need to remind the elderly in regular interval to complete the task; PA: Nurses need to partially assist elderly to complete the task, i.e. bring things near, help to carry heavy object, assist in holding things, etc. and FA: Nurses need to assist fully to complete the task.

| Subject | Assistance | Activity | Care Level |
| --- | --- | --- | --- |
| S1 | Independence | Meal | 3 |
|  | Check | Bath | 3 |
|  | Independence | Excretion | 3 |
|  | Independence | Move | 3 |
|  | Reminder | Dressup | 3 |
| S2 | Independence | Meal | 1 |
|  | Check | Bath | 1 |
|  | Check | Excretion | 1 |
|  | Independence | Move | 1 |
|  | Reminder | Dressup | 1 |
| S3 | Independence | Meal | 3 |
|  | Check | Bath | 3 |
|  | Check | Excretion | 3 |
|  | Independence | Move | 3 |
|  | Independence | Dressup | 3 |
| S4 | PA | Meal | 3 |
|  | PA | Bath | 3 |
|  | PA | Excretion | 3 |
|  | FA | Move | 3 |
|  | FA | Dressup | 3 |
| S5 | FA | Meal | 4 |
|  | FA | Bath | 4 |
|  | FA | Excretion | 4 |
|  | FA | Move | 4 |
|  | FA | Dressup | 4 |
| S6 | FA | Meal | 5 |
|  | FA | Bath | 5 |
|  | FA | Excretion | 5 |
|  | FA | Move | 5 |
|  | FA | Dressup | 5 |
| S7 | Independence | Meal | 3 |
|  | PA | Bath | 3 |
|  | PA | Excretion | 3 |
|  | FA | Move | 3 |
|  | FA | Dressup | 3 |
| S8 | Independence | Meal | 1 |
|  | Check | Bath | 1 |
|  | PA | Excretion | 1 |
|  | FA | Move | 1 |
|  | Independence | Dressup | 1 |
| S9 | Reminder | Meal | 3 |
|  | Check | Bath | 3 |
|  | PA | Excretion | 3 |
|  | FA | Move | 3 |
|  | Reminder | Dressup | 3 |
| S10 | Reminder | Meal | 1 |
|  | Check | Bath | 1 |
|  | PA | Excretion | 1 |
|  | PA | Move | 1 |
|  | Reminder | Dressup | 1 |
| S11 | Independence | Meal | 1 |
|  | Check | Bath | 1 |
|  | Independence | Excretion | 1 |
|  | Independence | Move | 1 |
|  | Reminder | Dressup | 1 |
| S12 | Independence | Meal | 3 |
|  | PA | Bath | 3 |
|  | PA | Excretion | 3 |
|  | FA | Move | 3 |
|  | PA | Dressup | 3 |
| S13 | Independence | Meal | 1 |
|  | Check | Bath | 1 |
|  | Independence | Excretion | 1 |
|  | Independence | Move | 1 |
|  | Independence | Dressup | 1 |
| S14 | FA | Meal | 5 |
|  | FA | Bath | 5 |
|  | FA | Excretion | 5 |
|  | FA | Move | 5 |
|  | FA | Dressup | 5 |
| S15 | Independence | Meal | 3 |
|  | Check | Bath | 3 |
|  | Independence | Excretion | 3 |
|  | Independence | Move | 3 |
|  | Reminder | Dressup | 3 |
| S16 | Independence | Meal | 3 |
|  | Check | Bath | 3 |
|  | Check | Excretion | 3 |
|  | Independence | Move | 3 |
|  | Independence | Dressup | 3 |
| S17 | FA | Meal | 4 |
|  | FA | Bath | 4 |
|  | FA | Excretion | 4 |
|  | FA | Move | 4 |
|  | FA | Dressup | 4 |
| S18 | Independence | Meal | 3 |
|  | PA | Bath | 3 |
|  | PA | Excretion | 3 |
|  | FA | Move | 3 |
|  | FA | Dressup | 3 |
| S19 | Reminder | Meal | 3 |
|  | Check | Bath | 3 |
|  | PA | Excretion | 3 |
|  | FA | Move | 3 |
|  | Reminder | Dressup | 3 |
| S20 | Independence | Meal | 1 |
|  | Check | Bath | 1 |
|  | Independence | Excretion | 1 |
|  | Independence | Move | 1 |
|  | Reminder | Dressup | 1 |
| S21 | Independence | Meal | 1 |
|  | Check | Bath | 1 |
|  | Independence | Excretion | 1 |
|  | Independence | Move | 1 |
|  | Independence | Dressup | 1 |
| S22 | FA | Meal | 5 |
|  | FA | Bath | 5 |
|  | FA | Excretion | 5 |
|  | FA | Move | 5 |
|  | FA | Dressup | 5 |
| S23 | Independence | Meal | 3 |
|  | PA | Bath | 3 |
|  | PA | Excretion | 3 |
|  | FA | Move | 3 |
|  | PA | Dressup | 3 |
| S24 | Reminder | Meal | 1 |
|  | Check | Bath | 1 |
|  | PA | Excretion | 1 |
|  | PA | Move | 1 |
|  | Reminder | Dressup | 1 |
| S25 | Independence | Meal | 1 |
|  | Check | Bath | 1 |
|  | PA | Excretion | 1 |
|  | FA | Move | 1 |
|  | Independence | Dressup | 1 |
| S26 | FA | Meal | 5 |
|  | FA | Bath | 5 |
|  | FA | Excretion | 5 |
|  | FA | Move | 5 |
|  | FA | Dressup | 5 |
| S27 | PA | Meal | 3 |
|  | PA | Bath | 3 |
|  | PA | Excretion | 3 |
|  | FA | Move | 3 |
|  | FA | Dressup | 3 |
| S28 | Independence | Meal | 1 |
|  | Check | Bath | 1 |
|  | Check | Excretion | 1 |
|  | Independence | Move | 1 |
|  | Reminder | Dressup | 1 |

## Data Set Room Layout:


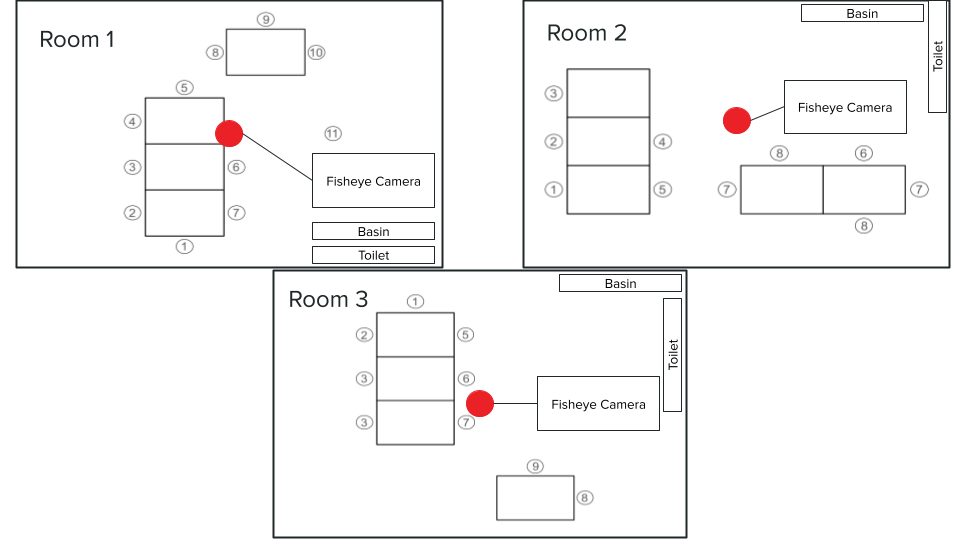


Figure S1. Full room layout for data set 1 and camera placement of each site. Here, Room 1: Site 1, Room 2: Site 2 and Room 3: Site 3. The fisheye camera is roof mounted.


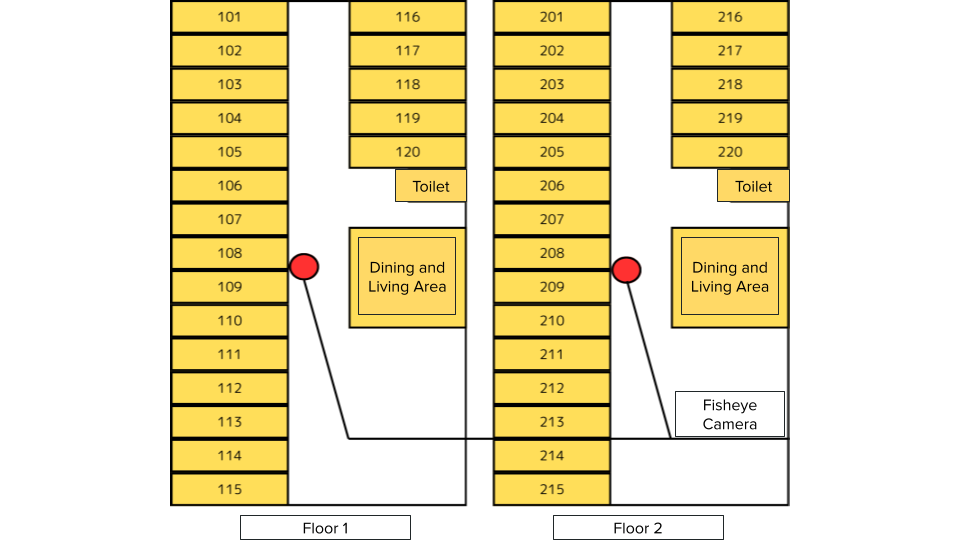


Figure S2. Data set 2 floor layout and camera placement of each site.
