# Supplementary material for: Integrating Care Context With Skeleton and Depth Information for Older Adult Activity Recognition in a Care Facility Using Care-Assessment-Aware Spatiotemporal Transformer: Method and Validation Study
Source: JMIR Aging. 2026 Apr 2;9:e80102. doi: 10.2196/80102 (PMC13087558; doi:10.2196/80102)
Supplement: Multimedia Appendix 2 [file aging_v9i1e80102_app2.docx]

Multimedia Appendix 3

Care Level Adding Process:

To add the care levels, we used a pose similarity test based on cosine dissimilarity scores, as our focus is on structural similarity. We established a cutoff for the low care group separation defined by the maximum cosine dissimilarity score observed within the low care level group in our data set. For a subject to be included as low care level group, their dissimilarity scores had to be below the following thresholds: eating <0.07, sitting <0.08, trying to stand up <0.11, and standing up <0.12. After identifying and separating the low care subjects, the remaining participants were carefully reviewed and assigned to mid care levels according to the established care scoring scale, while any uncertain cases were excluded from the dataset.

Toyota Smarthome Dataset:

- Number of participants: 17 (discarded 1 from the original data set 18)
- Age: 60-80
- Sensor: Kinect(7)
- Data: Skeleton
- Relabeled care level groups: Low (16) and Medium (1)

ETRI-Activity3D Dataset:

- Number of participants: 48 (discarded 2 from the original data set 50)
- Age: 64-88
- Sensor: RGBD camera
- Data: Skeleton and Depth
- Relabeled care level groups: Low (46) and Medium (2)

Performance Comparison:

Table S1: Dataset comparison. Here, P: Precision, R: Recall, F1: F1 Score, A: Accuracy.

| Data Set | P | R | F1 | A |
| --- | --- | --- | --- | --- |
| Toyota Smarthome | 0.91 | 0.93 | 0.92 | 0.92 |
| ETRI | 0.93 | 0.95 | 0.94 | 0.94 |
| Our Data | 0.97 | 0.96 | 0.96 | 0.96 |
